# Humanized Mice Are Instrumental to the Study of *Plasmodium falciparum* Infection

**DOI:** 10.3389/fimmu.2018.02550

**Published:** 2018-12-13

**Authors:** Rajeev K. Tyagi, Nikunj Tandel, Richa Deshpande, Robert W. Engelman, Satish D. Patel, Priyanka Tyagi

**Affiliations:** ^1^Division of Gastroenterology, Hepatology and Nutrition, Department of Medicine, Vanderbilt University Medical Center, Nashville, TN, United States; ^2^Biomedical parasitology Unit, Institute Pasteur, Paris, France; ^3^Department of Global Health, College of Public Health, University of South Florida, Tampa, FL, United States; ^4^Institute of Science, Nirma University, Ahmedabad, India; ^5^Department of Pediatrics, Pathology and Cell Biology, University of South Florida, Tampa, FL, United States; ^6^Zydus Research Centre, Ahmedabad, India; ^7^Department of Basic and Applied Sciences, School of Engineering, GD Goenka University, Gurgaon, India

**Keywords:** humanized/chimeric mice, malaria, NSG mice, TK/NOG mice, FRG mice, huRBCs, huHep, clodronate loaded liposomes

## Abstract

Research using humanized mice has advanced our knowledge and understanding of human haematopoiesis, non-adaptive and adaptive immunity, autoimmunity, infectious disease, cancer biology, and regenerative medicine. Challenges posed by the human-malaria parasite *Plasmodium falciparum* include its complex life cycle, the evolution of drug resistance against anti-malarials, poor diagnosis, and a lack of effective vaccines. Advancements in genetically engineered and immunodeficient mouse strains, have allowed for studies of the asexual blood stage, exoerythrocytic stage and the transition from liver-to-blood stage infection, in a single vertebrate host. This review discusses the process of “humanization” of various immunodeficient/transgenic strains and their contribution to translational biomedical research. Our work reviews the strategies employed to overcome the remaining-limitations of the developed human-mouse chimera(s).

## Background: the Burden of Malaria

Among the numerous infectious diseases, malaria remains a major health challenge. Malaria is a disease which spreads through the bite of female *anopheles* mosquitoes, who carry the infection moieties (sporozoites) of parasites belonging to the *Plasmodium* genus (1). The greater morbidity and mortality related to human malaria infections reported by World Health Organization (WHO), is due to the wide range of hosts, mainly affecting humans with a high host tropism. 216 million cases of malaria infection was reported across the world in 2016, with a death toll of 445,000. In comparison, 237 million cases were reported in 2010 and 211 million cases were reported in 2015 (2). The death incidence rate in Sub-Saharan Africa is more than 85% in children <5 years of age (3).

The pathophysiology and molecular mechanism of malaria has been revealed, contributing to improving our understanding of malaria biology. The routine culture of an asexual blood stage infection of *P. falciparum* was successfully achieved (4). However, various aspects related to the mechanism of cell motility and invasion, changes in cell signaling pathways and the modulation of host cells, escape from the immune system and establishment of an infection into the liver, or to stay in hypnozoite stages, are not very clear. The technological advances in the field of epidemiology and entomology support the research and reduces the burden of understanding the malaria parasitology by staining the vivid stages of all parasites (5–7).

The tens of millions of non-immune individuals from areas where malaria is not transmitted, visit malaria endemic areas, and face the risk of malaria infection (8, 9). The two major weapons against malaria are vector control and chemoprophylaxis/chemotherapy (10). Various treatments such as indoor residual spray (IRS), use of insecticide-treated bed nets, and medical care through antimalarial drugs (artemisinin-based combination therapy-ACT etc) are available to reduce the burden of malaria (11, 12). Unfortunately, attempts to eradicate the disease based on these methods have had only limited success due to wide spread drug resistance by the parasite (13) as well as insecticide resistance of the mosquito vector (14). This bleak situation drives scientist to develop additional control measures such as a malaria vaccine which is both appealing and urgent. However, an effective malaria vaccine development has not been found, despite enormous and continued efforts made in this direction (15).

### Biology of Malaria Parasites

**Figure 1:**
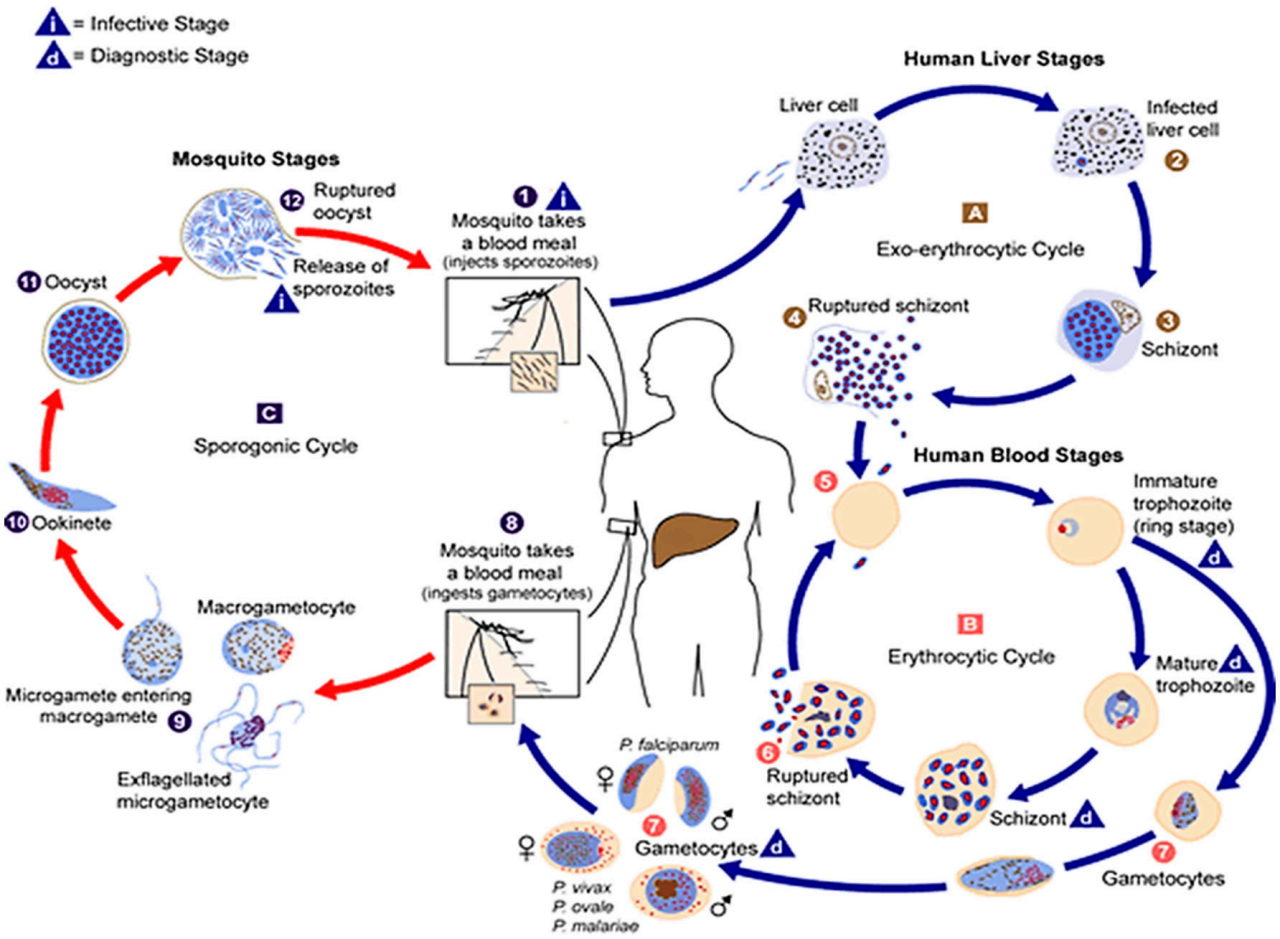
The malaria parasite life cycle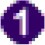. Sporozoites infect liver cells 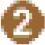 and mature into schizonts, which rupture and release merozoites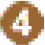. (Of note, in *P. vivax* and *P. ovale* a dormant stage [hypnozoites] can persist in the liver and cause relapses by invading the bloodstream weeks, or even years later.) After this initial replication in the liver (exo-erythrocyticschizogony 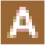), the parasites undergo asexual multiplication in the erythrocytes (erythrocyticschizogony 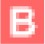). Merozoites infect red blood cells 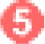]. The ring stage trophozoites mature into schizonts, which rupture releasing merozoites 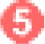]. Some parasites differentiate into sexual erythrocytic stages (gametocytes) 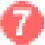. Blood stage parasites are responsible for the clinical manifestations of the disease. The gametocytes, male (microgametocytes) and female (macrogametocytes), are ingested by an *Anopheles* mosquito during a blood meal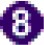. The parasites' multiplication in the mosquito is known as the sporogonic cycle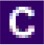. While in the mosquito's stomach, the microgametes penetrate the macrogametes generating zygotes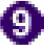. The zygotes in turn become motile and elongated (ookinetes) 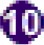 which invade the midgut wall of the mosquito where they develop into oocysts 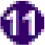. The oocysts grow, rupture, and release sporozoites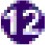, which make their way to the mosquito's salivary glands. Inoculation of the sporozoites 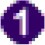 into a new human host perpetuates the malaria life cycle. (Source: https://www.cdc.gov/malaria/about/biology/index.html).

The causative agent of the malaria parasite has a complex multi-stage life cycle which commences with the bite of the female anopheles mosquito, a definitive host that carries sporozoites to infect healthy humans (intermediate host) to complete the life cycle of the parasite (Figure 1). The disease in humans is caused by one or a combination of *Plasmodium* spp.: *P. vivax, P. falciparum, P. malariae*, and *P. ovale* (7). Also, in geographically limited zones of South-East Asia, the Malaysian island of Borneo in particular, infections caused by *P. Knowlesi*, a zoonosis without visible transmission to other hosts, can be observed (16).

The systemic *P. falciparum* infection causes greater morbidity and mortality due to its severity and poor diagnosis. On the contrary, *P. vivax* infection is less severe, but formation of hypnozoites and their genesis, need to be studied to understand the mechanism of frequent relapses (17).

Malaria infection in humans begins when the sporozoites from the salivary glands of female *anopheles* mosquito enter the bloodstream via the skin and travel to the liver. The sporozoites traverse through blood vessels and reach the liver, and undergoes the tightly regulated signaling mechanism before rendering the infection to the hepatocytes (1). The invaded hepatocytes allow for the replication of sporozoites and leads to the formation of schizonts. This asexual reproduction stage stays inside the liver of human cells and lasts for 7–10 days, depending on the species and strain of the parasite. Each schizont gives rise to several merozoites which are released into the bloodstream, indicating the end of the exoerythrocytic phase of the malaria infection. The infection by *P. vivax* and *P. ovale* is not characterized with this reproduction step since pathogens might reside as hypnozoites within the hepatocytes for a longer duration, until the infection relapses (18).

The merozoites in the bloodstream invade the erythrocytes and the first stage after invasion is the formation of a ring that later evolves into a trophozoite. The trophozoite cannot metabolize heme so it converts heme into a yellow pigment, hemozoin. The globin part of hemoglobin is utilized as a source of amino acids, needed for reproduction. Trophozoite then develops into erythrocytic schizont and each mature erythrocytic schizont gives rise to new merozoites that eventually rupture the erythrocytes which are released into the bloodstream to invade new RBCs. This is the symptomatic stage in which clinical manifestations of the disease begin to appear. Further, unlike the liver stage, the erythrocytic stage of the malaria parasite repeats multiple times (7, 18).

The differentiation of the parasite into male and female non-pathogenic gametocytes (propagation carriers), also takes place within RBCs. If a female Anopheles mosquito takes a blood meal, the gametocytes are taken up and mature into macrogamete (female) and microgamete (male). The microgamete undergoes three divisions producing eight nuclei in the process in the mosquito's gut, and each nucleus fertilizes with a macrogamete resulting in the formation of an ookinete. The ookinete penetrates the midgut wall and becomes encapsulated, forming an oocyst. The ookinete nucleus divides, producing thousands of sporozoites within the oocyst. Toward the end of sporogony, lasting 8–15 days, the oocyst ruptures and sporozoites travel to the salivary gland of mosquito, ready to be injected into a human host during the next blood meal (7, 19).

## Human-Chimeric Mice (Humanized Mice)

Mice have been used as *in-vivo* models to study a diverse range of infectious pathogens (20). The murine and human genome shares nearly 85–99% similarity, but due to the complexity of the organization of cells, tissues and organs and specificity of the human immune system (21–23) some mouse strains are less than ideal *in-vivo* models. Recent advances made in translational biomedical research suggest the importance of immunomodulatory agents and target-specificity, and encourage the development of “humanized mouse models” (21, 24).

The theoretical and empirical evidence for the need of “human” mouse models, as well as advancements made in immunological research is the genesis and driving force for the development of chimeric mice. The immunodeficient mice transplanted with human cells or tissues are referred to as “humanized mice,” with engraftment and repopulation of a functional human system (25). Since tissue and cell grafts are perceived as foreign and rejected by the host immune system, immunodeficient mice are inevitably required to achieve human cell engraftment followed by their repopulation. Therefore, immunomodulatory agents are administered to modulate the immune response to reduce graft rejection episodes.

**Figure 2:**
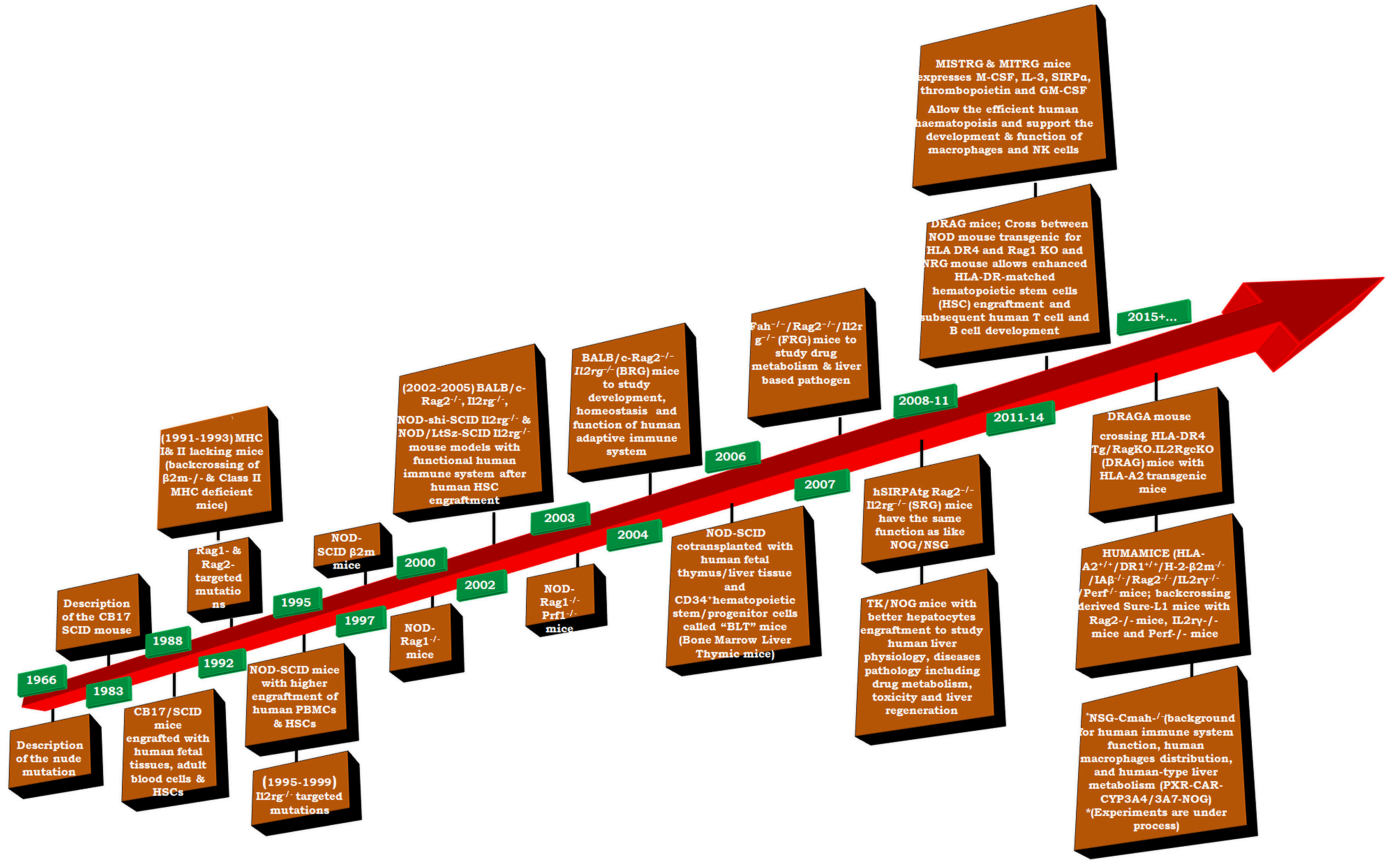
Timeline of important events in the development of humanized mice (adapted and modified from Shultz et al. (31).

Humanized-mice evolved from the origin of athymic (nude) mice with an underdeveloped/impaired thymus. The process of “humanization” of immunodeficient mice has progressed and a variety of immunodeficient mice have been developed by incorporating specific defects in their immune system through genetic engineering approaches. A brief account on immunological and physiological characteristics of some immunodeficient mice is given in Table 1 (26). The creation of human-mouse chimera(s) follows a cascade of different steps and events for the reconstitution of human cells, using various immunomodulatory protocols. All the procedures to prepare the host for human cells/tissue engraftment followed by their repopulation were described in detail (27), and have been reviewed many times (Figure 2) (21, 28–30) by multiple experts in the field.

**Table 1:** list of various immunodeficient mouse strains.

**Mice**	**Mutation**	**Mice Information**	**Character**	**Application**	**References**
BXN (Beige/Xid/Nude) Developed by the collaborative work of NIH, USA, and referred as “NIH-III”	Beige mutation (*bg*^J^ or *bg*^2J^)	Originally produced at Oak Ridge National Laboratory due to radiation-induced mutation, name represents the color of affected mice Available as a single mutation in C57BL/6J or C3H/HeJ Also available at Jackson Laboratory	Defective and reduced bactericidal activity, absence of NK cells and defects in CTLs and antibody responses to tumor cells Defect in lysosomal enzymes of neutrophils Short life span, low body weight and poor bleeding time	Identical mutant to Chediak-Higashi syndrome in humans which helps in understanding the similar abnormalities To study the role of pigment and their genes in combination with pale ear (*ep* mutation) Study of giant lysosomes an important marker in identification and studying hematopoietic stem cells Most importantly to study the mechanism of metastatic spread of cancer, infectious diseases, hypersensitivity, bone marrow grafts To distinguish between the cytotoxic function of NK cells and NK T cells in normal and beige mice	(32–39)
	Xid mutation	CBA/N mice having X-linked immune deficiency mutation Available at Jackson Laboratory	Depletion in the number of peripheral B-cells and The B cells having less surface IgM to IgD ration which represent the disorder in B-cell maturation Curtail in IgM and IgG3 concentration in the serum, and reduction in capacity (or inactivity) of thymo-independent MHC-II responses	Helpful in understanding the mutation of Bruton's tyrosine kinase gene (*Btk*) which plays an crucial role in B-cell development To study the expression of V_H_ gene in spleen Reveal the crucial role of IgM in preventing the infection of *Crytococccus neoformans* from lungs to the brain in chronic pulmonary infection model Role of BCR in TACI expression (T cell-independent type 1 Ag such as LPS which stimulate BCR)	(40–44)
	Nude mutation	Mutation in *FOX1* (winged-helix/forkead transcription factor) gene, also called athymic nude Abnormal hair growth First reported at Virus laboratory, Ruchill Hospital, Glasgow by Dr.N.R.Grist Available at Jackson Laboratory	Practical absence of the thymus hairlessness, lack of functional T-cells, and deficiencies in cellular immunity, Partial defect in B cell development However, there is no defect in T-cell precursors and therefore in adult mice some functional T cells can be observed Due to the defect in T helper cell activity, thymus-dependent antigen were detected by IgM only, high NK cell activity	As an experimental model for tumor inoculation to study the metastasis and their growth inhibition by drug treatment Suitable model to study the infectious diseases (*Mycobacterium leprae*, parasite infection of protozoa and worms) Studies of autoimmunity and allergy Model for the liver deficiency study and treatment and xenotransplantation	(40, 45–49)
SCID mice	Mutation in the *Prkdc^*SCID*^* gene on chromosome-16	Mutation in an enzyme which has an activity in DNA repair *Prkdc* (protein kinase, DNA activated, catalytic polypeptide) No V(D)J recombination occur *Prkdc^*SCID*^*naturally occurred in BALB/c-*Igh^*b*^* which are maintained at Institute of Cancer research in Philadelphia Available at Jackson Laboratory	Non-responsiveness of spleen toward T and B cells making them unavailable Ideal host for xenografting cells originating from the myeloid lineage remained unaffected due to their vital role for the survival which enable to generate lymphocytes bearing receptors of restricted diversity called “**leakiness**” Flawed DNA repair mechanismleave them susceptible toward radiation The first mouse strain that reportedly supported the human peripheral blood mononuclear cells (hu-PBMCs) engraftment to study HIV infection	Useful in studies of normal and abnormal lymphocyte development and function, role of nonlymphoid cells in the absence of lymphocytes Humanized SCID used to study the infectious diseases (HIV and EBV) and their therapeutics Suitable for the engraftment of human tissues such as tumor cells and cell lines, skin, pancreatic islets Ideal model for human tumor xenograft growth, pathophysiology and their metastasis Useful in studying the effect of radiation in normal and tumor cells for genetic deficiency and their repair mechanism	(50–54)
RAG mice	carrying a deleted recombination activating gene (*rag1* or *rag-2*)	Mutation in *Rag1* and *Rag2* gene which are important for V(D)J gene rearrangement that can produce the antigen receptor in B and T cells Available at Jackson Laboratory	Inhibits B-cell and T-cell differentiation, results into lack of B and T-cells NK-cell activity is prominent, they do not exhibit immunoglobulin leakiness and less sensitivity to radiation Shorter life span, produce higher amount of B cell lymphomas regularly	Commonly used as models to study the role of the immune system in cancer (tumorigenesis and metastases), autoimmunity, and chemotherapy studies as a potential alternative to Nude and *SCID* mice Higher level of engraftment of human HSC and lymphoid cells Study of the gene function in lymphocyte development by blastocyst complementation method	(55–58)
NOD mice	Contain a unique MHC haplotype (H-2^g7^), and a single nucleotide polymorphism (SNP) in the TNF-α and CTLA-4 genes	Non-obse Diabetic mice (NOD/ShiLtJ strain)	Impaired immune system with hampered maturation of macrophages, wound healing Defects in their natural killer (NK) cells and CD4^+^CD25^+^ T-cells Deficiencies of NK T-cells and absence of C5a complement	Important model for human type I diabetic study Allow the prolonged engraftment of human cells and tissues used for infectious disease and cancer Useful in the identification of responsible antigen, genes susceptibility for the diseases, effect of environment on the diseases, efficacy of therapeutics, and development of imaging techniques Humanized-NOD mice with higher susceptibility of vivid xenografts make it a suitable model for the translational biomedical research	(59–61)
NOD/SCID mice	Backcross of NOD with SCID mice (such as C3H/HeJ-SCID and C57/BL6-SCID) transferred the *Prkdc^*SCID*^* mutation	Developed by the Fox Chase Cancer center via transferring SCID mutation from C.B-17 to non-obese diabetic background Available at Taconic Bioscience/Jackson Laboratory	Unable to develop diabetes with diminished NK-cell activity and suppressed non-adaptive immune responses Capable of accepting a higher level of engraftment of hu-PBMCs, HSCs, liver and lungs No activity of complements and macrophages exhibiting the defects in the expression of cytokine receptors Shows 80-90% engraftment of HSCs in comparison to other strains Short life span of 8-9 months due to the severe thymic lymphomas, required special care and maintenance, paralysis may occur but changes are rare Leakiness is very less compare to other SCID models and chances of increases are less with the age	Usually used for translational biomedical applications and xenograft transplantation Recommended model for the study of the human platelet survival Study of the pathogenesis of drug induced immune thrombocytopenia (DITP) Useful in cancer biology research for the study of tumor formation, metastasis, their therapeutic treatment and regulation, imaging studies of tumor	(62–70)
NOD/SCID/β_2_m^−/−^ mice	Knockouts for β2-microglobulin gene which drives the expression and function of murine MHC-I molecules	Double homozygous for SCID and beta2m *B2m* mutation was developed by Dr. Beverly Koller and Dr. Oliver Smithies at University of North Carolina followed by *scid* and *B2m* combined mutation at Jackosn laboratory by Dr. Leonard Shultz	Bear the entire set of deficiencies prominent in the NOD/SCID mice along with complete death of NK-cell activity Disrupts the development and function of NK-cells, B and T-cells, this hampers the MHC-I-mediated immunity Several disadvantages for instance, capacity to sustain only limited development of human lymphocytes, restricted number of T-cells constituted and repopulated in the chimeric organs	Most suitable model for the xenograft transplantation (intra-bone marrow) To study the Influenza infection (due to restricted influenza-specific IgG), myelodysplastic syndrome For the vaccine development and the therapeutics for the tumor	(21, 71–74)
NOD/SCID/ IL2Rγ mice	Mutations in the *Il-2rg* gene	Referred as NSG mice NOD/schi-SCID Il-2rg^−/−^ (NSG) mice was developed by crossing the NOD/SCID mice with the *Il-2rg^−/−^* mice: *scid* and *IL2rg^*null*^* (SCID mutation in DNA repair complex mechanism and IL2 rg mutation prevent cytokine signaling via different receptors leading to deficiency in NK cell activity) Available at Jackson Laboratory	Impaired development and function of the lymphocytes and abolish NK-cell formation which in turn impedes IFN-γ secretion Absence of mature murine lymphocytes, functional dendritic cells, leakiness, and prolonged lifespan of mice for about 16 months (long-lived) Various immunodeficient mice were crossed with these IL-2rg^−/−^ knockouts to produce strains with further suppression of immunity	They exhibit excellent ability to accept human xenografts, especially PBMCs, stem cells, myeloid cells and progenitors originated from fetal liver, cord blood, bone marrow and PBMCs Exclusively used for the differentiation and development of HSCs, platelets, RBCs, and T-cells used for analyses Optimum mice model to study the mechanism of inflammation, wound healing, allograft transplantation and their rejection for the clinical purpose (specially human skin graft), remodeling of tissues and revascularization	(75–81)

*BCR, B-Cell receptor; TACI, transmembrane activator and calciummodulator and cyclophilin ligand interactor; LPS, lipopolysacchride; NK cells, Natural killer cells; CTLs, Cytotoxic T lymphocytes; MHC, Major histocompatibility Complex; hu-PBMC, Human peripheral blood mononuclear cells; HSC, Hematopoietic stem cells; RBCs, Red blood cells; SCID mice, Severe combined immunodeficiency mice; Rag1/2, Recombination activation gene 1/2; NOD, Non-obese diabetic*.

The developed mouse-human chimeras present an efficient pre-clinical *in-vivo* model to study the human immune system and its interaction with infectious pathogens, hematopoiesis, stem cell development and function, tumor formation, cancer biology, and regenerative medicine.

### Human-Mouse Chimeras Required to Study Infectious Pathogens

The pathogens relevant to human disease, which do not infect other animal species, required an animal model that could reconstitute or harbor human tissues to replicate the human immune system (82). The study of human infectious diseases is restricted to *in-vitro* models as humanization of immunocompromised mice is difficult, due to the scarce data available on host-pathogen interaction, cell behavioral pattern and the biological complexity of the human body. The *in-vitro* effect of drugs and therapeutic effect in animals differ in their metabolic pathways, and does not give an accurate prediction of drug metabolic pathways for humans (83). Drug metabolism in humans and their physiological differences in animal model(s) also make it arduous to design and formulate the efficient drugs and their targets. The development of disease-specific drugs will be possible only when the pathogenicity as well as molecular and immune mechanisms of infectious pathogen is well understood. The testing of preclinical drugs and candidate vaccines require humanized mice that help predict the drug metabolism and pharmacokinetics.

Humanized mice have been used over a long period of time, which helps understand the mechanism of rejection of human tissues provoked by human immune cells. However, translation of these studies from murine (immunodeficient mice) models to the clinic, has limited success due to numerous reasons which demand precious focus on human immune responses to allogeneic tissues along with their cumbersome usages and required skilled personnel (84). The technology for cell and tissue transplantation has improved the reconstitution of human immune cells/tissue that allows for the successful study of graft vs. host diseases (GVHD), different allograft studies and other human immune system studies which have been established as a robust preclinical model for human-specific therapeutic inventions (30, 81, 84). The human immune system may be engrafted into the immunodeficient mice, where type of human cells [pluripotent stem cells and their derived cell populations, regulatory T cells (Treg)] and tissues (human skin, islet, cardiac tissues, xenografts) are important in engraftment (81). Therefore, a robust small laboratory animal model transplanted with human hematolymphoid/hematopoietic stem cells is indeed required to advance drug discovery (85).

The liver, an important immunogenic organ, consists of innate immune cells [natural killer (NK) cells, NK-like T cells, Kupffer cells and dendritic cells (DCs)] which play an important role in the local immune surveillance, liver regeneration and pathogenesis of liver diseases (86–88). The enzymes involved in the drug and xenobiotic metabolism and excretion, liver has been the center for the research related to drug mechanism and identifying novel drugs. Hepatotropic viruses and parasitic infections are the cause for a greater rate of mortality in humans, in addition to the toxicity rendered by chemicals. Humanized mice are therefore useful in order to study viral infections with hepatitis B or C and the hepatic stage of the malaria (89) infection. Acute liver failure becomes a predominant issue because of the drug-induced liver injury (90) and uncharacterized behavior of the drug metabolic pathway and clearance from the human body. Therefore, human liver reconstituted immunodeficient mice have been deployed to study the drug metabolic pathways (90, 91). Several approaches have been deployed, including the *in-vitro* culture of human cell lines, transgenic insertions of human genes, cultures of human hepatocytes and chimeric mice with the repopulated humane liver. The *in-vitro* culture has the limitation of continuity which cannot delineate the human liver metabolism. Despite several advantages, expression of transgene, inhibitor and activator proteins of the cells and cofactors required for the reactions derived from the mouse, are some of the limitations (89). To overcome these limitations, investigators have employed human hepatocytes in the culture to investigate the human-relevant drug metabolism processes (89, 92–95). Earlier studies confirmed that hepatocytes are important for the immune tolerogenic properties of the liver (96,–98) and their transplantation inhibits allograft rejection (99). Although results obtained by the human hepatocytes with respect to the drug metabolism, studies were limited to the short duration, a maximum of 30–40 days (100, 101). Recently, transgenic mice (TK/NOG) conditioned with ganciclovir (GCV) treatment have shown to deplete a number of mouse hepatocytes to create receptive stroma for the injectable human hepatocytes (huHep). huHep reconstituted humanized mice have been used to test the drugs to predict their pharmacological and toxic nature on human liver (83, 102). It was later observed that human chemokine production by the engrafted huHep is one of the mechanisms that improve the human immune systems reconstitution in the liver of LTH hu-mice (human FTHY and CD34^+^ HSPCs) (103) since they do not, or respond poorly to mouse counterparts (104, 105). These results indicate the improved human immune system reconstitution in the liver of LTH hu-mice (103).

Humanized mice are used to study different diseases, which have been reviewed several times (20, 25, 29–31, 74, 106–109) along with necessary modifications to overcome the limitation of respective models (24, 70, 81).Therefore we did not give a detailed account on mouse models developed to study other diseases, and focused only on the humanized mouse model(s) used to study the biology of *P.falciparum*.

## Humanized Mice are Instrumental to Study the *P. falciparum*

Advancements made in biomedical research are not sufficient to fight systemic inflammatory diseases such as *P. falciparum* malaria infection in humans. The mouse-human chimeras have been used to study the asexual blood stage, exo-eryhrocytic stages (liver stage), and transition from liver-to-blood stage (17, 110–113) infection of *P. falciparum*. However, specific immune-responses against antigens produced in all stages of the parasite are yet to be explored (114, 115). And, the stage specificity of human parasites does not allow them to grow and develop in murine models (116).

Rodent parasites (*P. berghei* and *P. yoelii*) are used as surrogates to study the pre-erythrocytic infection of human malaria parasites. The species mismatch between the rodent and human parasites makes it difficult to extrapolate (116) their data for human studies. However, there are syntenic proteins such as hepatocytes surface protein CD81 which helps to render the infection in a rodent parasite and *P. falciparum* (117). Similarly, a sporozoites surface protein named thrombospondin-related anonymous protein TRAP is required for liver infection in the human malaria parasite (118) and rodent parasite (*P. berghei*) (119). On the other hand, hepatocytes growth factor receptor (C-Met) plays an important role in the early pre-erythrocytic infection of the liver by *P. berghei* (120). This receptor is not important to study the biology of *P. yoeli* or *P. falciparum* (121). Likewise, a merozoite adhesive erythrocytic protein MAEBL is required for the liver stage infection in *P. falciparum* by sporozoites (122) whereas it is not required in case of *P. berghei* (123). The human malaria parasites were never seen to infect the murine hepatocytes (112). Therefore, a humanized mouse is required to model *P. falciparum* to understand its basic biology, pathophysiology, immunology and pharmacology.

The pre-erythrocytic stage of the parasite life cycle is asymptomatic, and all clinical pathological symptoms are shown because of the progression of the asexual blood stage infection. The erythrocytic stage is routinely studied *in-vitro* by the development of a continuous culture system that allows the replication and growth of an asexual blood stage parasite within human RBCs (4, 124). Recent advancements in the *in-vitro* culture of *P. falciparum* has expanded the knowledge of its blood stage to show the basic parasitology (5) and the development of target specific drugs effective against asexual blood stage infection (125).

The sporogonic stages are generated by feeding the female *Anophele*s mosquitoes on *in-vitro* gametocyte cultures, and the progression of the parasite life cycle in the mosquito as well as subsequent sporozoite accumulation in the salivary gland of the mosquito is allowed. However, the *P. falciparum* liver stage (LS) has proven much more difficult to study even though complete LS development in primary huHep and subsequent transition to *in-vitro* erythrocytic infection was shown over 30 years ago (126) followed by subsequent studies (118, 127–129). The primary human hepatocytes, or HepG2 cells, have been used to study the development of LS infection of *P. vivax* (130, 131). FRG-KO huHep mice were used to study the LS infection and formation of hypnozoites during the *P.vivax* infection (132).

Currently, drug resistance against the *Plasmodium* strains remains the main hurdle in effective vaccine development. However, there is remarkable improvement in the development of novel drug therapies. On the other hand, development of novel tools are a prerequisite of the rapid transition of preclinical drugs to the clinic, along with the fulfillment of SERCaP (Single Exposure Radical Cure and Prophylaxis) guidelines (133). To address the above-mentioned issues, recently developed humanized mice helped investigating the different stages of malaria parasites in detail (6).

### Humanized Mice Support the Development and Replication of Asexual Blood Stage Infection of *P. falciparum* (RBC Reconstituted Humanized Mice)

The human-red blood cells (huRBCs) host the asexual blood stage infection of *P. falciparum*. The huRBCs reconstituted immunodeficient NSG mice present nearly an ideal model to develop an understanding of the asexual blood stage infection. The different strains of immunodeficient mice, which were used for the asexual blood stage infection, are listed in Table 2. Different strategies provided a humanized environment for the circulation of human immune effecters and huRBCs, maintaining the stable long-term blood chimerism (17). The sizeable and long-term huRBCs grafting (human blood chimerization) and control of inflammatory responses provoked by the parasites are crucial factors contributing to the success of humanized mouse (134). The same humanized mouse (PfhuRBC-NSG-IV) was used to experimentally induce the artesunate resistance and to validate the resistance phenotype (135).

**Table 2:** The developed “humanized mouse model(s)” for asexual blood stage infection of *P. falciparum*.

***P. falciparum* strain(s)**	**Humanized mice**	**Applications**	**References**
NF54 or multiresistant T24	BXN (Beige/Xid/Nude)	First rodent model for *P.falciparum* study, to test the novel compounds for the drug development and evaluation of drug responses, vaccine development	(136–138)
FUP, NF54, 3D7, Dd2 and clinical isolates	SCID (Severe Combined Immunodeficiency) and NOD/SCID	Helpful in *in-vivo* studies of human malaria parasites and vaccine development	(139–141)
Mouse-adapted 3D7	NOD/SCID/β_2_microglobulin (β_2_m)^−/−^	*in-vivo* experimental drug/exposure-response assay	(142)
Mouse-adapted 3D7	NOD/SCID/IL-2 receptor γ chain (IL2Rγ)^null^or NSG mice	To check the response against various antimalarial therapeutics	(143, 144)
Different parasite strains (3D7,UPA and K1) without any prior adaptation	NOD/SCID/IL-2 receptor γ chain (IL2Rγ)^null^ or NSG mice with an additional treatment of clodronate delivered through liposomes	Most comprehensive humanized mouse model allowing to develop the sexual stage parasites besides the asexual-blood stage development	(134, 145)
NF54, 3D7	HLA-transgenic mice	Discovery of novel protective malaria antigen and immune responses	(146)
NF54	TK/NOG	Used to assess the drug toxicology and metabolism, and *P. falciparum* infection in transplanted human hepatocytes	(6)

*TK/NOG, thymidine kinase/NOD/Shi-scid/IL-2Rγnull mice; HLA, human leukocytes antigen; 3D7, NF54, PAM, different P. falciparum strain*.

#### Other Blood Stage Humanized Mouse Models

Tsuji et al. first reported the transplantation of huRBCs into immunodeficient SCID mice (140). To overcome the rapid elimination of huRBCs from the mouse's periphery, they discovered that the intraperitoneal (IP) injection of human serum significantly extended the survival of huRBCs injected intravenously (IV). After the successful and stable engraftment of huRBCs into SCID mice, some of the animals were irradiated (dose of 300 cGray) 2 days prior to *P. falciparum* infection (FUV strain) and a blood smear was drawn for the Giemsa staining. They observed the rapid elimination of infected and non-infected huRBCs from the non-irradiated SCID mice, compared to the irradiated mice with a decrease in the parasitemia during the initial 5 days of infection. The trend of parasitemia remained constant even upon dilution, by administering huRBCs up to 20 days. This report suggested that *P. falciparum* could grow and develop only in huRBCs (140). However, the SCID mice needed further modifications to establish the hosting of huRBCs. This mouse proved to be a valuable tool for the *in-vivo* study as well as the vaccine development of human malaria parasites.

Similarly, Moore *et al* used the NOD/SCID mice infected with NF54 and 3D7 *P. falciparum* strains, with a single dose of uninfected huRBCs, injected intraperitoneally to see their development in the mouse's circulation. Later, infected and un-infected huRBCs were injected intrapertioneally every day. They observed the stable human blood chimerization and parasitemia in the peripheral blood for 16 days post-infection. Also, the sexual form of parasites (gametocytes) were observed and assessed with respect to their infectivity (139). In conclusion, NOD/SCID mice could also serve as a model for humanization without using any immunomodulatory agent. This might help in understanding the role of the immune response against the *P. falciparum* infection, but the adaptation of parasites and ascetic solutions prior to IP injection was required.

Later, Badell *et al* suggested the use of AB^+^ huRBCs to achieve a significant blood chimerism in SCID/NIH III mice for extended periods. However, they used different immunomodulatory agents to deplete the host's residual innate immune effecters. The dichloromethyleneblphosphonate-(CI_2_MBP)-encapsulated liposomes were administered weekly intraperitoneally. The parasitemia was estimated up to 3% and maintained over 0.3% for 17 days. All the developmental stages of asexual erythrocytic cycle were observed. Therefore, this mouse model was used to study the drug(s) at pre-clinical levels for the development of a vaccine (136) and to assess the effect of antimalarial drugs reported for human infection (138). These findings overcame the limitations seen with previous models deploying novel immunomodulation protocols.

To develop an ideal mouse model to carry out wider applications of *P. falciparum*, Sabeter *et al* used CIMP (chemical immunomodulation protocol) in NOD/SCID mice to see whether they are susceptible and receptive to infection. They compared their model with the existing huRBCs-NIH III (BXN) mouse. The CIMP was modified and infected huRBCs, with various stages of parasites injected into the NOD/SCID mice intraperitoneally and followed until the end of the study. PCR and Giemsa staining were used to determine the low-grade parasitaemia together with a histopathological analysis. The infected percentage of NOD/SCID and BXN was estimated at 75 and 8%, respectively for 35 days post-infection. The higher-grade parasitemia in BXN and initial lower parasitemia was the hall mark in NOD/SCID for 7 days. The greater rise in the parasitaemia was seen during the second infectious challenge on day 17. PCR was performed on the DNA extracted from the animals, which showed 100% sensitivity and 67.3% specificity. The histology on different organs such as the bone marrow, spleen, liver, lungs and kidney was carried out. The malaria pigments (MP) inside the phagocytic cells were responsible for the elimination of infected-huRBCs, from the peripheral blood used as an indicator. These results suggested the distribution of MP laden macrophages were dependent on the parasitaemia and mouse strain (149). In brief, treatment of CIMP showed the better engraftment of huRBCs in NOD/SCID mice and supported the development and progression of *P. falciparum* infection. NOD/SCID mice were used to test the anti-malarial activity of novel compounds. The susceptibility of NOD/SCID mice with two different *P. falciparum* strains (3D7 and Dd2) and clinical isolates were assessed using slightly modified immunomodulation protocols. Results showed the parasitaemia of 0.05–8% sustained for 19 days with the development of asexual and sexual stage of parasites. These findings suggest that prior adaptation of parasite to its host is not necessarily required for the growth and development of the parasite (141).

#### Recent Development and Currently Available Humanized Mice for Asexual Blood Stage

The usage of chemical agents may influence the parasites development as well as the unexplored interaction with antimalarial drugs. Thus, without the use of any additional immunosuppressive agents, the successfully developed NOD/SCID/β_2_microglobulin (β_2_m)^−/−^ mice paved the way for the asexual stage of *P. falciparum* to examine the antimalarial compounds and the human response (142).

The mouse-adapted *P. falciparum* strain was used to study the infectious challenge in a novel mouse model “NSG mice” (NOD/SCID/IL-2 receptor γ chain (IL2Rγ)^null^). These mice supported the ten-fold higher burden of parasitized and non-parasitized huRBCs, compared to the NOD/SCID/β_2_microglobulin (β_2_m)^−/−^mice (144). Although administration of huRBCs intraperitoneally presents better engraftment (142, 143), the transperitoneal passage of blood from the peritoneum to the blood stream was one of the major issues observed with the IP model (134). The value of this mouse model was validated by the assessment of the therapeutic potential of antimalarial drugs (147).

The optimal blood stage mouse model was developed by employing different malaria parasite strains, without requiring prior adaptation to the host. The huRBCs injection and infectious challenge were administered through intraperitoneally and intravenously (134, 145), respectively. The high rising and long-standing parasitaemia was achieved in the mouse by controlling the number of monocytes/macrophages through the treatment of clo-lip (Supplementary Figure 1). This pharmacological agent was shown (145) to deplete 70–80% murine monocytes/macrophages. The model also demonstrated a higher level of parasite synchronization and partial sequestration of tainted huRBCs in the vasculature, a hallmark marvel observed in malaria patients. The huRBCs reconstituted mouse showing sustained and stable development of asexual blood stage parasites, also supports the development of sexual stages (gametocytes) (Supplementary Figure 2) (145). The developed humanized mice was used to induce the experimental induction of high level artesunate resistance in *P. falciparum* and to validate the resistance phenotype (135).

The huRBCs reconstituted NSG mice were challenged with *P. falciparum*, showing the development of gametocytes. Further, the developed mouse model allowed the *in-vivo* experimental drug/exposure-response assay (147). Developed humanized mice, harboring asexual blood stages of *P. falciparum*, more closely resembled the process in humans. Later, huRBCs-NSG mice were used to understand the interaction of gametocytes with the bone marrow and spleen and the efficacy of anti-gametocytidal activity of drug(s). The value of this mouse model was validated by assessing the efficacy of primaquine, which kills the sexual stages of *P. falciparum*. The clearance of gametocytes, compared to that seen in the control, suggests that the immunomodulation protocol does not hinder the assessment activity of drug effectiveness against the transmission stage of the *P. falciparum* infection. This helps better understand the role of drugs on the gametocytes present in the sequestration sites (150).

The supply of human serum and hypoxanthine resulted in the augmentation of a greater huRBCs chimeric index and parasitic growth. The continuous supply of huRBCs with an effective immunomodulatory protocol is inevitable for the effective and successful growth and development of the *P.falciparum* infection in available humanized mice. The issue of poor huRBCs grafting was overcome by the administration of human cytokines (151). The gamma irradiated NSG mice were injected with CD34^+^ HSC intracardially and showed more than 40% human leukocytes reconstitution administered with the plasmid containing erythropoietin and human IL-3 intravenously (152). Mice showing at least 1.5% huRBCs reconstitutions were used for the subsequent experiments. Purified mature schizonts (3D7 strain) of the *P. falciparum* (*ex-vivo* infection) were added to the blood, maintained in RPMI 1640, supplemented with serum and incubated at 37°C for up-to 64 hrs, followed by the Giemsa staining. Within 16 hrs of infection, ring stages were observed with the parasitaemia of 0.02 to 1.6%. The infected-RBCs harvested from the humanized mice were analyzed by fluorescent activated cell sorting. The infectious challenge in *de-novo* synthesized and reconstituted humanized mice were detected by microscopy, PCR and flow cytometry. This shows the variation in the infection ability of various strains tested. The *P. falciparum* infection in the *de -novo* generated huRBCs as well as re-invasion was observed (151).

#### Sexual Stages and Blood-to-Mosquito Transmission

To determine the infectivity of the developed sexual parasites in huRBC-reconstituted NOD/SCID mice, *Anopheles stephensi* and *A. freeborni* mosquitoes directly fed on mice showing sexual stages. The oocyst formation in the mid-gut of the mosquitoes 7–10 days post-feeding, indicates the infectivity of sexual parasites and their successful transmission from humanized mice to mosquitoes (139). Similarly, the FRG-NOD huHep mice successfully produced the asexual blood stage parasites when they were challenged with NF54 *P. falciparum* infection. Subsequently, parasites were maintained in a continuous culture and produced gametocytes. The mosquitoes fed on these sexual stages, and upon dissection, the number of oocyst and sporozoites were estimated as the same in the salivary glands of infected mosquitoes (111). Later, the sexual parasites developed in other humanized DRAG mice were investigated for the transmission to mosquitoes via direct infection or through an *in-vitro* culture method. The development of oocyst and sporozoites in *A. stephensi* mosquitoes in both conditions was observed (146). Further, TK/NOG mice showed the development of all stages of gametocytes and maintained it for a longer duration. However, their transmission to mosquitoes required further experimental validation (6).

### Liver-Humanized Mice to Study Exoerythrocytic (EE)/Liver Stage Infection of *P. falciparum*

From the multistage life cycle of the human malaria parasite in their primary (mosquitoes) and secondary host (human), the least known stage is the exoerythrocytic (EE)/liver stage. Notably, the exploration of the LS infection of *P. falciparum* in a human host is hampered by the low success rate of a hepatic stage culture, which is limited to a few days and which can be extended up to a month (128). This is possible with *P. falciparum*, by *in-vitro* human hepatocytes alone or through the co-culture (129, 153) in HC-04 cells, but with very low sporozoites infectivity estimated at 0.066% with *P. falciparum*, posing a challenge to study LS infection (127). The infectivity of sporozoites were later seen to increase up-to 0.8% (122, 154) and 1.3–1.4% in the same hepatocytes cells (HC-04) during the study of protein O-fucosylation activity (118), which is still relatively low compared to what could be achieved with primary hepatocytes or with *P. berghei/yoelii* infection in *in-vitro*. Therefore, the development of the more convenient *in-vivo* model will help better understand the liver pathophysiology within a human host. A transgenic and immunodeficient mouse strain showing better control over its non-adaptive immune response is therefore needed. The chimeric mouse model(s), as reported elsewhere (109), facilitates the understanding of LS and mimics humans to develop a further understanding of the various biological phenomena of LS parasites.

#### Liver Stage Mouse Models: Developments and Recent Advances

Following this direction, the first attempt was made to transplant the huHep in SCID mice (110). huHep were transplanted through open mouse survival surgery, followed by an infectious challenge of infected sporozoites isolated from the *A. stephensi* mosquitoes. The histopathological studies were carried out on sections of various tissues removed on day 3, 7, and 9 post-infectious challenge. Immunofluorescence assays were carried out with circumsporozoite protein (CSP), merozoite surface antigen 1 (MSA-1) and liver stage antigen-1 (LSA-1) to detect the *P. falciparum* infection in transplanted hepatocytes. The success rate of transplanted huHep-SCID mice were estimated at around 95% and infection was detected even 4 months after human hepatocyte transplantation. The results obtained were similar to that of non-human primates such as chimpanzees. However, the rate of sporozoites infection and their correlation with other existing animal models need to be evaluated (110).

Liver with severe chronic disease conditions, can enhance the engraftment of huHep (155). The expression of uPA was back-crossed with SCID or SCID/Beige background which resulted in the better engraftment and repopulation of huHep, measured up to 14 weeks at regular interval post-transplantation. The proteomic analysis was performed to evaluate whether the huHep were functioning properly through COFRADIC^TM^. uPA/SCID-huHep mice were given the infectious challenge with the *P. falciparum* sporozoites, and the liver of infected animals was extracted for immunofluorescence assay to carry out a gene expression study. Several specific antibodies, reported for parasites, were used to detect the *P. falciparum* infection. Furthermore, RNA was extracted from the liver tissues to see the expression of specific genes such as PfCSP, PfLSA-1, and PfMSP-1, present during the early and late liver stage infection (156). The longer period of survival of parasites within the transplanted huHep were questioned. Therefore, residual innate immune effecters were controlled by the IP injection of clodronate-liposome. Immunohistochemical studies have shown higher engraftment of human hepatocytes within the clusters of hepatocytes. The intravenously injected sporozoites were seen to infect huHep as well as the development of schizonts on day 5 in the sections made from liver tissue and stained with the Hematoxylin-Eosin stain. The use of different antibodies and gene expression studies confirmed the development of liver stage infection (157). The role of sporozoites-expressed genes P52 and P36 (important for the hepatocyte infection) in genetically attenuated parasites (GAPs) showed the development (158) and expression of liver stage antigen-1 (LSA-1) during the *P. falciparum* infection (159) in uPA/SCID humanized mice. Also, uPA/SCID humanized mice were used to better understand the migration of sporozoites inside the human host and to study the escape mechanism of SPECT (sporozoites microneme protein essential for cell traversal) and PLP1 (perforin-like protein 1) employed by *P. falciparum* sporozoites (154). However, the poor breeding efficiency, a limited time-window for transplantation, renal diseases in reconstituted mice (160) and liver injury caused by the plasminogen treatment which leads to the other complications, were major concerns (148). Additionally, mice were not used to study the transition of parasite from liver-to-blood stage infection in one host (147).

Grompe et al developed a model with a mutation in the fumarylacetoacetate hydrolase (FAH) enzyme. These mice however, died within 12 hrs of birth, due to liver complications (161). Therefore, these FAH-KO needed continuous drug (NTBC) pressure to avoid liver damage and associated health complications (162). Thereafter, several mouse models were developed by back-crossing FAH knockout mice with NOD/SCID or RAG1. None were able to repopulate the human hepatocytes. Besides, FAH/nude, and FAH/RAG1 were also unable to engraft the human cells due to the immune rejection (160). Nevertheless, FAH/NOD/SCID mice were grafted with a smaller number of human cells. However, liver failure resulted in the death of animals when NTBC pressure was withdrawn (160).

#### Liver Stage Mouse Model and Transition From Liver-to-Blood Stage Infection

As Rag2^−/−^*/Il2rg*^−/−^ mice have shown the highest engraftment, FAH^−/−^ were backcrossed with Rag 2 and IL2Rγ and resulted in the triple knock-out (KO) of FRG mice (FAH^−/−^Rag2^−/−^IL2Rγ^null^). This mouse model achieved more than 90% engraftment of huHep and several mice survived up to 4 months, even after the withdrawal of the NTBC drug, which was confirmed by histological, immunocytochmeical and RT-PCR analysis specific to human hepatocytes (160). The FRG-huHep chimeric mice were injected with *P. falciparum* sporozoites intravenously. Mice were euthanized on day 3, 5, 6, and 7 post-infection for histopathology, indirect immunofluorescence assays (IFAs) and gene expression studies. Results of all the studies confirmed the successful infection in transplanted huHep, transcripts of *P. falciparum*, which amplified specific genes, and the development of LS infection (111). The formation of mature merozoites followed by the transition to the blood-stage is a very important step, as it allows for the study of a complete life cycle in one host. C57BL/6 background of the FRG mice hindered the engraftment of huRBCs because of incompatibility with SIRPα (163). Therefore, FRG, and NOD/SCID mice were back-crossed to create “FRG-NOD” which was reconstituted with huHep and challenged with *P. falciparum* sporozoites followed by intravenous injection of huRBCs on the 6^th^ and 7^th^ day post LS infectious challenge. The huHep reconstituted mice showed the development of LS infection (111). Subsequently, blood was processed with an *in-vitro P. falciparum* culture medium to cultivate the parasites. The blood smears were drawn from the culture and stained with Giemsa solution every 24 h. The gametocytes along with different asexual stages of parasites were observed. This indicates the successful transition from liver-to-blood-stage infection (111).

FRG-huHep mice were used to assess the humoral immune response against the LS infection of *P. falciparum* (164). The natural infection route (mosquito's bites) was chosen, rather than adopting the conventional route of administration of sporozoites. FRG-huHep mice have revealed the role of inhibitory antibodies against the *P. falciparum* infection. This humanized mouse model could be used to optimize and assess the efficacy of candidate vaccines (164) prior to the clinical trials. Recently, FRG-huHep mice were used to determine the anti-liver stage potential of Atovaquone-Proguanil and Primaquine (165). A novel antimalarial molecule (DSM265), which reportedly selectively targets *Plasmodial* dihydroorotate dehydrogenease (DHODH) *in-vitro* and *in-vivo* blood stage and improve liver stage activity in *in-vitro*, was tested in these mice (166). The oral dose of the drug quantified the log reduction to 2.5 in the liver parasites by *in-vivo* bioluminescent imaging through an IVIS spectrum. The obtained results helped to understand the mechanism of action, the pharmacokinetic or pharmacodynamic study of drugs, before deploying to clinical trials (165).

Likewise, FRG-huHep mice with slight modifications in the administration of huRBCs were used, with the additional treatment of the cytotoxic chemotherapy agent cyclophosphamide with clo-lip, to prevent the phagocytosis of huRBCs (167). This revealed the effective long terms survival of transplanted mice, allowed the infection in huHep, as well as the higher number of infected huRBCs on day 7 post-sporozoite infection challenge, a successful liver to blood stage transition and a sustained asexual blood stage infection of *P. falciparum* with increasing parasitemia. The *P. falciparum* reticulocyte-binding protein homolog 5 (RH5) is an important merozoite invasion ligand which interacts with huRBCs and is essential for the infection of all the parasite strains (168). Therefore, the anti-RH5 antibody in FRG-huHep/huRBC mice were used to determine its efficacy and utility. qRT-PCR, IVIS and parasite multiplication rates (PMRs) were observed to determine the potential of blocking the blood stage infection *in-vivo* while transitioning from the liver stage (167). The FRG mice, despite allowing the successful development of liver and blood stages, and despite being helpful in the assessment of the efficacy of various novel candidate vaccines and drugs, need skilled personnel to maintain the higher level of huRBCs on day 6 post-LS challenge (167).

#### Next Generation Mouse Models for Exoerythrocytic Stage and Completion of Life Cycle in One Host

To overcome the limitations of uPA/SCID and FRG mice, a transgene called herpes simplex virus type 1 thymidine kinase (HSVtk) was expressed in the hepatocytes of NOG mice (TK/NOG). The host was prepared by GCV conditioning which induces the apoptosis of liver cells to deplete the number of murine hepatocytes. The reconstitution of GCV conditioned TK/NOG mice show the sizeable huHep-repopulation index (huHep-RI) confirmed by the immunohistochemical staining of different antibodies. The value of developing human liver chimeric (huHep-TK/NOG) mice was assessed by determining the efficacy of human specific drug metabolism in these mice. The long-term survival (more than 6 months) without any exogenous drug treatment justifies the value of huHep-TK/NOG to study the LS infection of *P. falciparum*. TK/NOG mice allow the additional dose of GCV even after the human cell repopulates, which helps achieving higher re-population index of human xenografts (148). Additionally, TK/NOG mice hardly require additional drug supplements to suppress the host immune system, except the residual cells of the monocyte-macrophage lineage (169). huHep reconstituted TK/NOG mice favor and support the development of LS and transition to blood stage infection.

**Figure 3:**
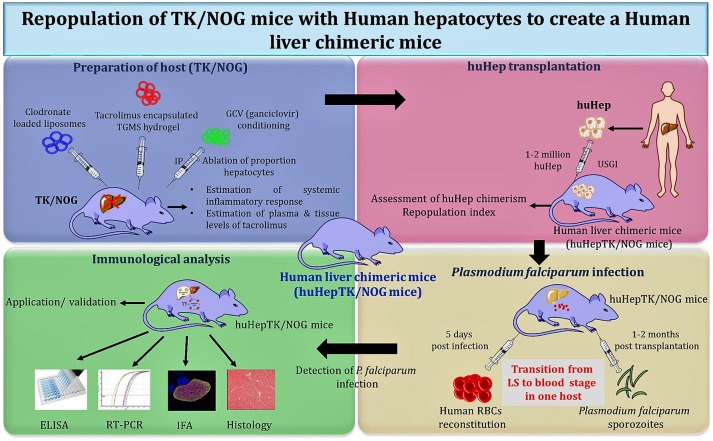
Schema of “creation” for human liver chimeric mice to study LS infection of *P. falciparum* and subsequent transition to blood-stage infection.

Studies were carried out with the huHep-TK/NOG mice to gauge the metabolism of anti-inflammatory drug (diclofenac) in humans (170). These mice revealed the potential for reliable 2-fold engraftment, for all phases of the *P. falciparum* life cycle within one host. huHep-TK/NOG mice supported the huRBCs reconstitution at days 5–6 post sporozoite challenge in the same host, which allowed for the transition from LS–to blood-stage infection of *P. falciparum* (Figure 3) to be studied. The added advantage with this mouse model, unlike others (111), is that it does not need two mouse strains to get the complete life cycle in one host.

Soulard V *et al*. and his group used the TK/NOG mice to study the LS infection of *P. falciparum* (6). huHep-TK/NOG mice were injected with sporozoites intravenously and liver sections extracted from the infected mice were analyzed on the 5^th^ and 7^th^ day post-infection. The parasite development was confirmed specifically in the huHep region. huHep reconstituted mice receiving LS infection when administered with huRBCs intraperitoneally daily, reached 50–60% blood chimerism within the 6^th^ day and 80–90% on day 12. On the 6^th^ day post-LS infection, the transition from the liver to blood stage in the same host was observed. The blood sample was collected and maintained in *in-vitro* culture for 8–10 days to reveal the *P. falciparum* blood stage parasites. The aggregate gametocyte numbers could constitute up to 8.5% of total parasites, and 2.5% mature gametocytes (6). It was revealed that the TK/NOG model, most closely resembles human processes and is therefore the most suitable for preclinical drug studies and *in-vivo* LS infection.

Various humanized mouse models used to study liver-stage infection is summarized in Table 3 (147).

**Table 3:** The comparative analysis of different LS humanized mice to study inflammatory diseases, (Adapted and modified from Vaughan et al. (147).

		**Alb-uPA SCID (Beige)**	**FAH^**−/−**^Rag2^**−/−**^IL2R**γ^null^** (FRG)**	**TK/NOG**	**HLA-DR4.RagKO.IL2Rγ** **cKO.NOD (DRAG)**
Liver Injury	Mutation	uPA (urokinase-type plasminogen activator) overexpression	Fumarylacetoacetate hydrolase deficiency	Herpes simplex virus type 1 thymidine kinase (HSVtk) transgene expression	NRG mice chimeric with human-mouse class II transgenes encoding the HLA-DR4 genotype fused to the *I-E^*d*^* MHC class II molecule
	Occurrence	At birth	At birth and increased by the NTBC treatment	On day 7 and 5 prio to transplantation, liver cells treated with GCV and maintain without any exogenous drug pressure	At birth
Transplantation age		Within 3 weeks of post-birth	Any (adult)	Adult 8-week-old	Four months of post infusion of human HSC
Human Chimerism		up to 100%	up to 90%	more than 90%	High (human cells and HSCs) but human RBCs were less (0.2–1%)
Throughput		Low	Medium	High	Medium
Additional challenges		Continuing and progressive damage to liver parenchymal stage, poor breeding efficiency, renal disease	Development liver carcinomas, under drug exposure for longer duration	No systemic morbidity	Less number of human hepatocytes developments, develop a functional human immune system, development of mouse monocytes
*Plasmodium falciparum*	Sporozoite infection	Yes	Yes	Yes	Yes
	**Liver stage development**				
	Early	✓	✓	✓	✓
	Mid	✓	✓	✓	✓
	Late (merozoite release)	✓	✓	✓	✓
	Liver-to-blood stage transition	No	Yes	Yes	Yes
Erythrocyte co-engraftment possible		No	No	Yes	Yes (low)
Unique advantage		No	No	Additional GCV treatment is used twice only prior to huHep transplantation	Fusion with HSC develop human hepatocytes, kupffer cells, liver endothelial cells and erythrocytes, Sustain complete life cycle of *P. falciparum* without exogenous addition of human hepatocytes/RBCs
Stability and reproducibility of humanized liver		human chimerism can only be achieved in homozygous SCID/ Alb-uPA immunodeficient recipients	Long life span through NTBC drug treatment, but other associated complications are seen	Over the 8 months of pro-long period without any drug pressure	Human RBCs can detect for up to 4 months
References		(147)	(147)	(6, 148)	(146)

*NTBC, 2-(2-nitro-4-(trifluoromethyl)benzoyl)cyclohexane-1,3-dione; GCV, ganciclovir*.

#### Human-Immune System Repopulated Mouse Models to Study the Liver Stage Infection

Humanized mice with a human immune system (HIS) will help to develop pre-clinical models for the study of infectious diseases. Mice with the HIS expression called DRAG (HLA-DR4.RagKO.IL2RγcKO.NOD) were employed to study the complete life cycle of *P. falciparum* when transplanted with CD34^+^ HSCs (146). After 4 months post-transplantation with human HSC, DRAG mice were challenged with *P. falciparum* sporozoites. The parasitaemia was measured by PCR as well as counted on the Giemsa stained blood smears. The liver sections were observed 5 days later by using immunohistochemistry for HSP70. The blood stage parasites were observed between 10 and 28 days post infectious challenge, followed by the blood stage *in-vitro* culture wherein rings, trophozoites and schizonts were observed (146). The complete life cycle of *P. falciparum* in one host was achieved, but low parasitaemia levels, the development of a functional human immune system and host monocytes (in DRAG mice) and adaptation of the parasite strain(s), are concerns which need to be addressed (6). The recently developed humanized DRAGA mice were injected with the liver *P. falciparum* sporozoites (Pfspz) along with Chloroquine diphosphate pressure (171) to elicit the pre-erythrocytic immunity. These animals were protected against the challenge with infectious Pfspz, but had no protection against the asexual blood stage infection (171). We believe this humice could serve as a pre-clinical tool to study the immunogenic potential of newly discovered malaria candidate vaccines and drugs.

Very recently, NSG mice were transplanted with primed human spleen cells (hu-Spl-NSG) to assess the immune response and to understand the biology of the pathogen, to develop an effective vaccine (172). The two different constructions of the liver stage antigen-3 (LSA-3) (shorter and longer) which express during the liver stage and on the surface of sporozoites used as anti-LSA-3, may prevent the invasion of hepatocytes by sporozoites and protect them. Both constrains of protein have shown the different results, as the shorter form of LSA-3 shows the production of IFN-γ (humoral immune response) which was null in the full length protein, whereas the full length of LSA-3 protein showed the production of T regulatory (Treg) cells, but not a shorter constrain. The similarity of Treg sequences found in the human samples was confirmed. hu-Spl-NSG mice might be used to estimate the immune response, and overcome the limitations of graft vs. host reactions seen in hu-PBMCs reconstituted mice (172).

The complete cycle of (LS to blood stage transition) *P. falciparum* in developed humanized mice, will help performing numerous applications for vaccine development and formal genetic studies. In conclusion, developed human liver chimeric mice will revolutionize translational biomedical research and open doors for the vaccine development and drug therapeutics for other inflammatory diseases such as hepatitis, dengue, cancer, AIDS, and TB.

## Conclusion and Future Perspectives

Considering the importance of humanized mice in biomedical research, coordinated efforts are being made to engineer the advanced immunocompromised mice, to support human cell (HSCs and hepatocytes) grafting. The introduction of genetic immunodeficiency in mice plays a central role during “humanization” to reduce graft rejection episodes. Therefore, various mouse strains have been engineered to deplete the host's immunity, which aims to achieve sizeable grafting of human cells/tissues. A major contribution of these mouse model(s) is to study the erythrocytic stage and liver stage infection of *P. falciparum*. We believe an ideal humanized mouse model will be instrumental to study novel drugs and their targets, assessing the immunogenicity of novel candidate vaccines. Higher immunosuppression and novel transplantation strategies are indeed needed to create a small laboratory, straightforward and reproducible humanized mouse model. This review will help the development of new methods to create a humanized mouse with stable human cell transplantation and repopulation, to study systemic inflammatory diseases.

## Author Contributions

RT conceived the idea of the study, carried out all experiments related to mouse humanization and modeling *P. falciparum* in humanized mice and wrote the manuscript. NT and RD helped format and write the manuscript. RE reviewed the manuscript. SP provided logistics. PT helped in the preparations of figures and tables and writing of the manuscript.

### Conflict of Interest Statement

The authors declare that the research was conducted in the absence of any commercial or financial relationships that could be construed as a potential conflict of interest.

## Abbreviations

ACT: Artemisinin-based combination therapy
AIDS: acquired immune deficiency syndrome
anti-Gr1: Anti-granulocyte receptor 1
APCs: antigen presenting cells
cGy: centigray (equals to 0.01 gray)
CIMP: Chemical immunomodulation protocol
Clo-lip: Clodronate-loaded liposomes
CYPs: Cytochrom P450s
DCs: Dendritic cells
FAH: fumarylacetoacetate hydrolase
*FoxN1*: forkhead Box N1
FRG: FAH^−/−^Rag2^−/−^IL2Rγ^null^
GCV: ganciclovir
GM-CSF: Granulocyte-macrophage colony-stimulating factor
GVHD: graft versus host disease
hBMCs: human bone marrow cells
HepG2: human liver carcinoma cells
hESCs: Human embryonic stem cells
hPBLs: human peripheral blood lymphocytes
HSCs: Human hematopoietic stem cells
HSVtk: herpes simplex virus type 1 thymidine kinase
huBMCs: Human bone marrow cells
huHep: Human hepatocytes
huPBLs: Human peripheral blood lymphocytes
huRBCs: Human-red blood cells
IFA: immunofluorescence assay
IFN-γ: interferon gamma
*IL-2rg*: interleukin-2 Receptor ɤ-chain
IP: Intra-peritoneal
iPSC: Induced pluripotent stem cells
IRS: indoor residual spray
IV: Intravenous
KO: knockout
LS: Liver stage
MCP-1: Monocyte chemoattractant protein-1
M-CSF: Macrophage colony-stimulating factor
MP: Malaria pigments
MST: Median survival time
NICC: Neonatal porcine islet cell clusters
NK-cell: Natural killer cells
NOD: Non-obese diabetic
PBMCs: Peripheral-blood mononuclear cells
PHA: Phyto-haemagglutinin
*Prkdc*^*SCID*^: Protein kinase, DNA activated, catalytic polypeptide; Severe Combined Immunodeficiency
qRT-PCR: Qualitative reverse transcriptase polymerase chain reaction
*Rag*1/2: Recombination-activating gene 1/2
RBCs/huRBCs: Red blood cells/human-red blood cells
RH5: Reticulocyte-binding protein homolog 5
rhGH: Recombinant human growth hormone
rhuPRL: Recombinant human prolactin
RI/huHep-RI: Repopulation index/huHep-repopulation index
SCID: Severe combined immunodeficiency
SIRPα: Signal regulatory protein alpha
TB: Tubercuosis
TCR: T-cell receptor
TNF-α: Tumor necrosis factor-alpha
TRAP: Thrombospondin-related anonymous protein
Tregs: Regulatory T cells
uPA: Urokinase plasminogen activator
USGI: ultra-sound guided injection
WHO: World Health Organization.
