# Supplementary material for: Humanized Mice Are Instrumental to the Study of Plasmodium falciparum Infection
Source: Front Immunol. 2018 Dec 13;9:2550. doi: 10.3389/fimmu.2018.02550 (PMC6315153; doi:10.3389/fimmu.2018.02550)
Supplement: Supplementary file 1 [file Data_Sheet_1.docx]

Supplementary Material

***Humanized Mice Are Instrumental To The Study Of Plasmodium Falciparum Infection***

Rajeev K.Tyagi^#*^, Nikunj Tandel^#^, Richa Deshpande, Robert W. Engelman, Satish D. Patel and Priyanka Tyagi

# Co-first authors

***Address for correspondence**

**Rajeev K. Tyagi, Ph.D.** Email: [rajeev.tyagi@vumc.org](mailto:rajeev.tyagi@vumc.org), [rajeev.gru@gmail.com](mailto:rajeev.gru@gmail.com)

**Section 1: Mouse model to study asexual blood stage infection of *P.falciparum***

Our group tried the intraperitoneal (IP) route to administer human RBCs as well as infectious challenge. However, issues of transperitoneal passage of huRBCs from peritoneum to the blood stream led us switch to intravenous route to inoculate the infected and uninfected huRBCs (Supplementary Figure 1) (Tyagi et al., 2011)***.***

The huRBCs reconstituted immunodeficient mouse show significant human blood chimerism leading to the successful engraftment of *P. falciparum* parasite. The NSG mouse supported not only the infected and uninfected huRBCs engraftment (Supplementary Figure 2 A, B, C, D, E & F) but also the development of sexual stages (propagation carriers) of *P. falciparum* (Supplementary Figure2 G, H)*.* The developed humanized mouse harboring asexual blood stages of *P. falciparum* is the only model which is closer to the human situation. The partial sequestration and development of gametocytes (propagation carriers) are well-supported by *P. falciparum* infected with blood stage parasites. Although we tried to feed mosquitoes on Pf-huRBC/NSG mouse harbored with gametocytes in their circulation, infectivity of sporozoites could not be established (Tyagi et al., 2011).

**Figure Captions**

**Supplementary Figure 1**: Development of mouse model for *P. falciparum* engraftment (PfhuRBC/NSG-IV) mouse, A) The level of successful engraftment of human RBCs into mouse against the number of days the animal administered huRBCs with, B) The parasite load in huRBC reconstituted NSG mice. The PAM, 3D7 & K1 are geographically different *P. falciparum* laboratory strains The NSG mice supported the development of all laboratory strains, C) synchronization: the pattern of *P. falciparum* growth over the period of time. The arrows indicate the points during which >95% parasites were found in the ring stage

**Supplementary Figure 2:** Different stages of parasites observed in NSG-IV mice. Panel A & B were shown the ring stage followed by fully mature trophozoites after 24 hrs in panel C. Panel D illustrates the highest parasitemia during the phagocytosis by monocytes. E & F panel represent the fully developed schizonts regularly seen in peripheral blood. Panel G & H panel rarely saw the mature gametocytes (stage IV & V).

**References**

Tyagi, R.K., Arnold, L., Meija, P., Swetman, C., Gleeson, J., Pérignon, J.-L., and Druilhe, P. (2011). Further improvements of the P. falciparum humanized mouse model. *PloS one* 6**,** e18045.
