# Supplementary figures and images for: Humanized Mice Are Instrumental to the Study of Plasmodium falciparum Infection
Source: Front Immunol. 2018 Dec 13;9:2550. doi: 10.3389/fimmu.2018.02550 (PMC6315153; doi:10.3389/fimmu.2018.02550)

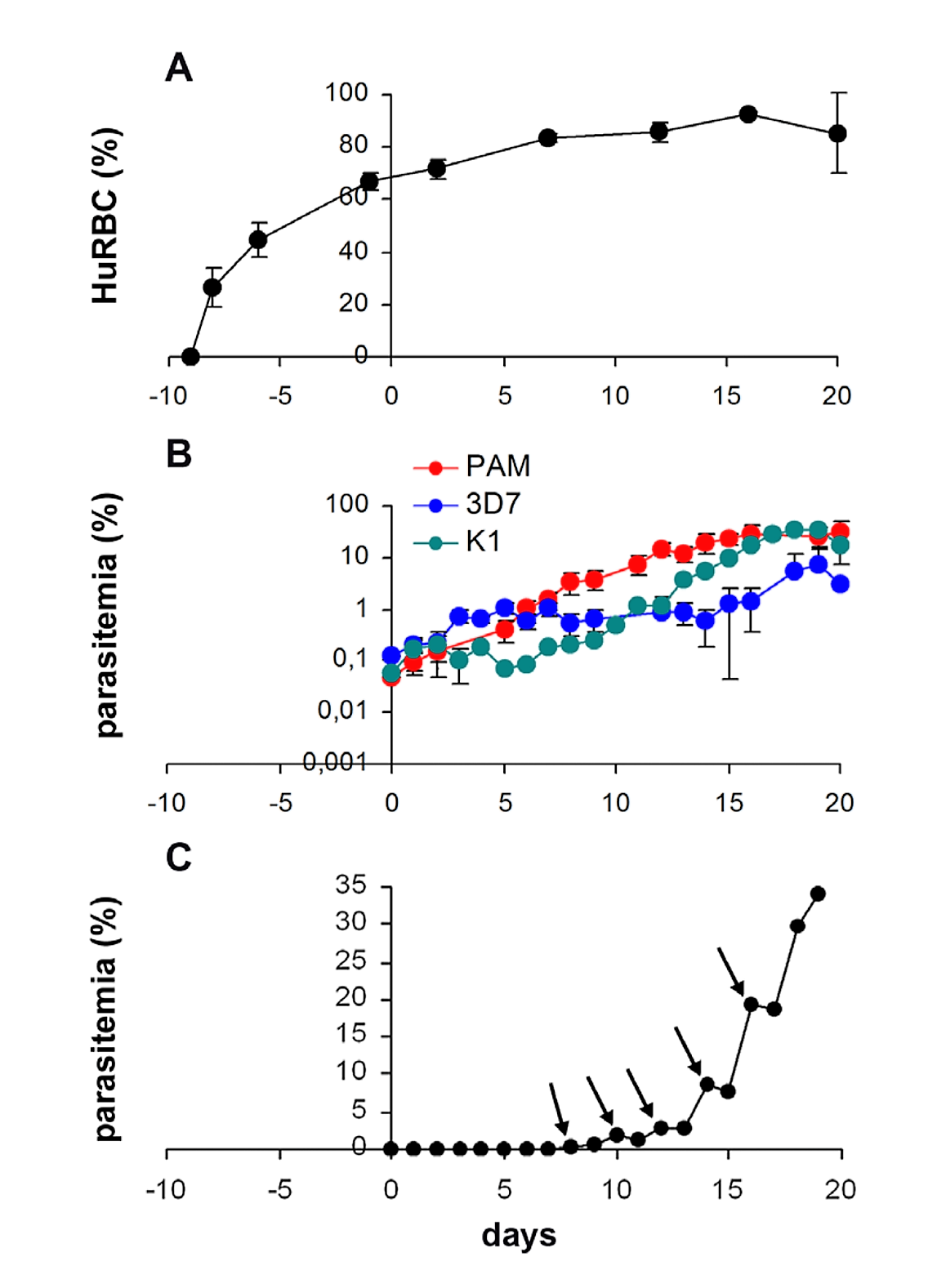

Supplement: Supplementary file 2 [file Image_1.tif]

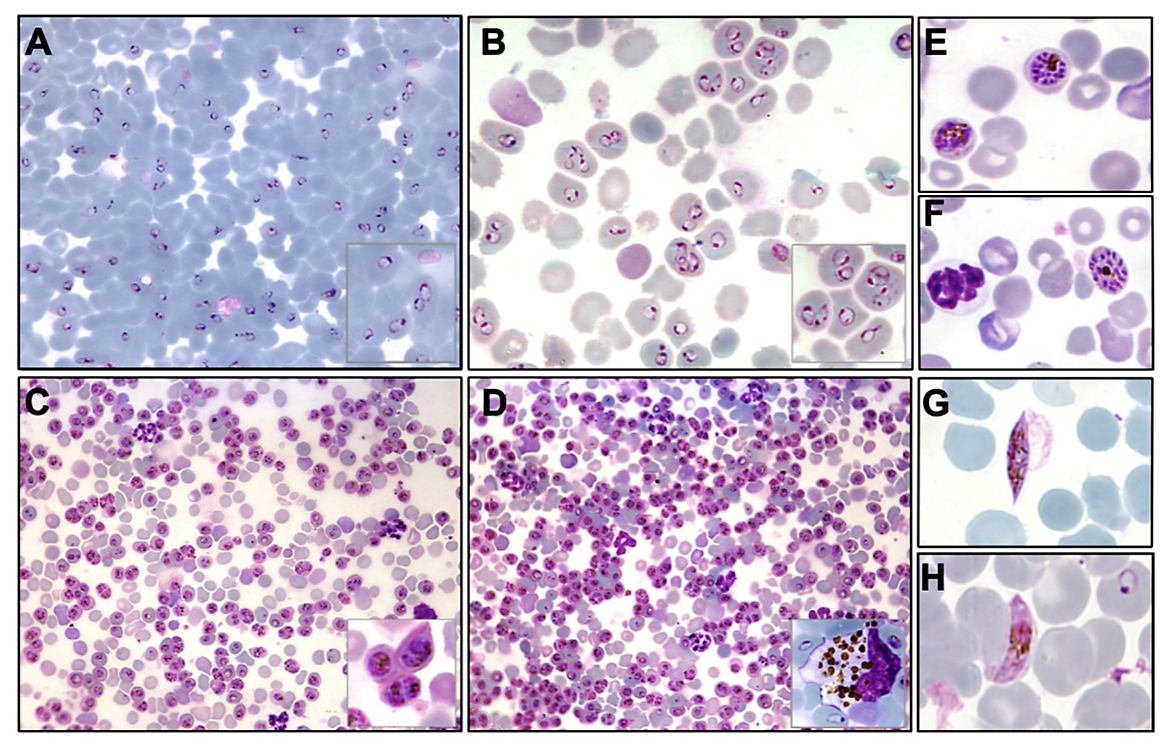

Supplement: Supplementary file 3 [file Image_2.tif]
